# Update of the molar incisor hypomineralization: Würzburg concept

**DOI:** 10.1007/s40368-023-00848-5

**Published:** 2023-10-19

**Authors:** K. Bekes, R. Steffen, N. Krämer

**Affiliations:** 1 Department of Paediatric Dentistry, University Clinic of Dentistry, Medical University of Vienna, 1090 Vienna, Austria; 2 Private Practice, Weinfelden, Switzerland; 3 Department of Paediatric Dentistry, Justus Liebig University, Giessen, Germany

**Keywords:** Molar incisor hypomineralization, MIH, Würzburg concept, MIH Treatment Need Index, MIH-TNI, Treatment plan

## Abstract

**Purpose:**

Molar incisor hypomineralization (MIH) is playing an increasingly important role in dental practice. MIH is defined as hypomineralization of systemic origin of one to four permanent first molars, often associated with affected incisors. Affected teeth are more susceptible to caries and post-eruptive enamel loss and should be diagnosed and treated as early as possible. In 2016, the Würzburg concept was developed for German-speaking countries including a classification index—the MIH Treatment Need Index (MIH-TNI)—and a treatment plan based on it for the use in daily practice. In the meantime, the concept has also gained international recognition. The aim of this paper is to update part 2 of the Würzburg concept, the treatment plan, as knowledge about MIH has increased and the disease has been studied more extensively in the last years. Other treatment approaches are now available and therefore need to be included in the concept. Although, the evidence of the different treatment options is still weak, practitioners need guidance in their daily practice.

**Methods:**

The authors reviewed the available literature, including clinical and laboratory studies and published guidelines.

**Results:**

The updated version of the Würzburg concept includes additional non-invasive strategies and temporary therapy options, as well as treatment approaches for incisors. It therefore covers currently available treatment modalities for MIH-affected teeth, ranging from prophylaxis, non-invasive treatment to restorative approaches and possibly even extraction.

**Conclusions:**

This is intended to help guide the practitioner and will need to be further validated by clinical trials.

## Introduction

Molar incisor hypomineralization (MIH) stands as a perplexing and increasingly prevalent dental condition that has gained significant attention within the field of (paediatric) dentistry and oral health research. First described in the 1980s (Koch et al. 1987), the term was coined in 2001 by Weerheijm et al. (Weerheijm et al. 2001). MIH is characterised by a qualitative deficiency in enamel mineralization, predominantly affecting the permanent first molars with or without the involvement of incisors. The aetiology still remains unclear although several systemic and genetic and/or epigenetic factors acting synergistically or additively seem to be associated with MIH, revealing a multifactorial aetiology model (Garot et al. 2022). The average prevalence worldwide is 13.1–14.2% (Schwendicke et al. 2018; Zhao et al. 2018; Schwendicke et al. 2019).

MIH presents a considerable clinical challenge due to its diverse clinical spectrum. The severity of enamel defects can range from mild opacities with minimal functional impact to extensive post-eruptive breakdown, and increased sensitivity leading to structural compromise and significant discomfort and making affected teeth susceptible to caries and dental pain (Weerheijm 2004; Lygidakis 2010). In general, the darker the colour of the opacity, the softer and more porous the enamel (Marouane and Manton 2022), and the greater the risk of posterior substance loss (usually at the cusps) with exposure of dentin (Weerheijm et al. 2003).

For the diagnosis of MIH, the criteria proposed by the EAPD are internationally well known and established. They take into account the specific clinical signs and symptoms of the disease: demarcated opacities, post-eruptive enamel breakdowns, atypical restorations and extractions of molars (Weerheijm 2003; Lygidakis et al. 2022; Somani et al. 2022). In addition, affected teeth can be further classified into mild defects and severe defects (Lygidakis et al. 2022; Somani et al. 2022).

## Development of the Würzburg concept

In 2016, the Würzburg concept was developed by a working group with representatives from Germany, Austria and Switzerland during the spring conference of the German Society of Paediatric Dentistry (DGKiZ) (Bekes et al. 2016, Bekes and Steffen 2016, Steffen et al. 2017) and has since gained increasing international acceptance (Hahn et al. 2020; Butera et al. 2022; Joshi et al. 2022; Olczak-Kowalczyk et al. 2023). The concept includes a classification index—the MIH Treatment Need Index (MIH-TNI)—and a treatment plan based on it. The idea for the concept was based on the fact that at that time almost all available classifications described in the literature mostly used the defect as a criterion, ignoring the possible combined presence of sensitivity, which is clinically relevant. In addition, most of them were not linked to a specific treatment recommendation (Lygidakis et al. 2010). The EAPD's updated 'Best Practice Guidance', published in 2022, has now also filled this gap (Lygidakis et al. 2022). Overall, the Würzburg concept should help guide practitioners in their daily practice, although the evidence for different treatment options is still weak.

## Part 1: MIH treatment need index (MIH-TNI)

The MIH-TNI captures the clinical key symptoms of MIH (Bekes and Steffen 2016; Steffen et al. 2017). It includes the presence and the extent of the breakdown and the problem of hypersensitivity. A total of four different grades of MIH can be distinguished (Table 1), depending on the presence/absence of breakdown and hypersensitivity. The index can be applied to all teeth and is not restricted to permanent teeth or individual groups of teeth. It is suitable for use and study in larger populations as well as for accurate description of findings in individual patients (Bekes and Steffen 2016; Steffen et al. 2017). The MIH-TNI has also been tested for its psychometric properties. It has been found to be valid (Stratigaki et al. 2020; Pflugi 2021).

**Table 1:** MIH Treatment Need Index (MIH-TNI)

Index	Definition
Index 0	No MIH, clinically sound
Index 1	MIH: without breakdown, without hypersensitivity
Index 2 2a 2b 2c	MIH: with breakdown, without hypersensitivity extension of defect < 1/3 extension of defect ≥ 1/3 to < 2/3 extension of defect ≥ 2/3 or/and defect close to the pulp or extraction or atypical restoration
Index 3	MIH without breakdown, with hypersensitivity
Index 4 4a 4b 4c	MIH with breakdown, with hypersensitivity extension of defect < 1/3 extension of defect ≥ 1/3 to < 2/3 extension of defect ≥ 2/3 or/and defect close to the pulp or extraction or atypical restoration

## Part 2: treatment plan 1.0

Based on the MIH-TNI, a therapy plan in form of a flow chart was developed in a second step (Bekes et al. 2016). In 2016, this was the first MIH concept to provide both—a classification index and a therapy plan based on the index. Clinicians who do not treat MIH everyday are often uncertain how to deal with affected children. However, it is important for patients to receive the right comprehensive care at an early stage. Depending on the severity of the hypomineralization, the therapy to be favoured ranges from intensive prophylaxis to restorative measures or even extraction. Of course, these available options are well known. Nevertheless, uncertainty in choosing the "right" therapy often causes problems for general dentists. The choice of treatment option depends on a number of factors. These include the severity of MIH, the presence of symptoms, the age of the patient, and the social background and expectations of the child and the parents (Lygidakis et al. 2022; Somani et al. 2022). The first step must always be an early diagnosis, which should be accompanied by prophylactic measures as soon as possible.

The aim of the treatment plan developed for the Würzburg concept was to guide clinicians in their daily work by providing an easy-to-use flow chart. The treatment approaches included the sections of prophylaxis, regeneration, sealing, immediate treatment, and long-term planning. Since individual treatment options must be seen in relation to the caries risk of the patient, two structurally identical flow charts were created: one for patients with low caries risk and one for patients with high caries risk.

The structure of the flow chart was as follows: In the first horizontal row, the four indices were shown. In the first column all available treatment approaches were displayed: prophylaxis (at home, in office), sealing, temporary restoration (short-term), temporary restoration (long-term), permanent restoration and extraction. The flow chart should be read in such a way that after the diagnosis (MIH-TNI 1-4), the user can find the treatment options in the appropriate column.

## Update of the Würzburg concept: version 2.0

**Fig. 1:**
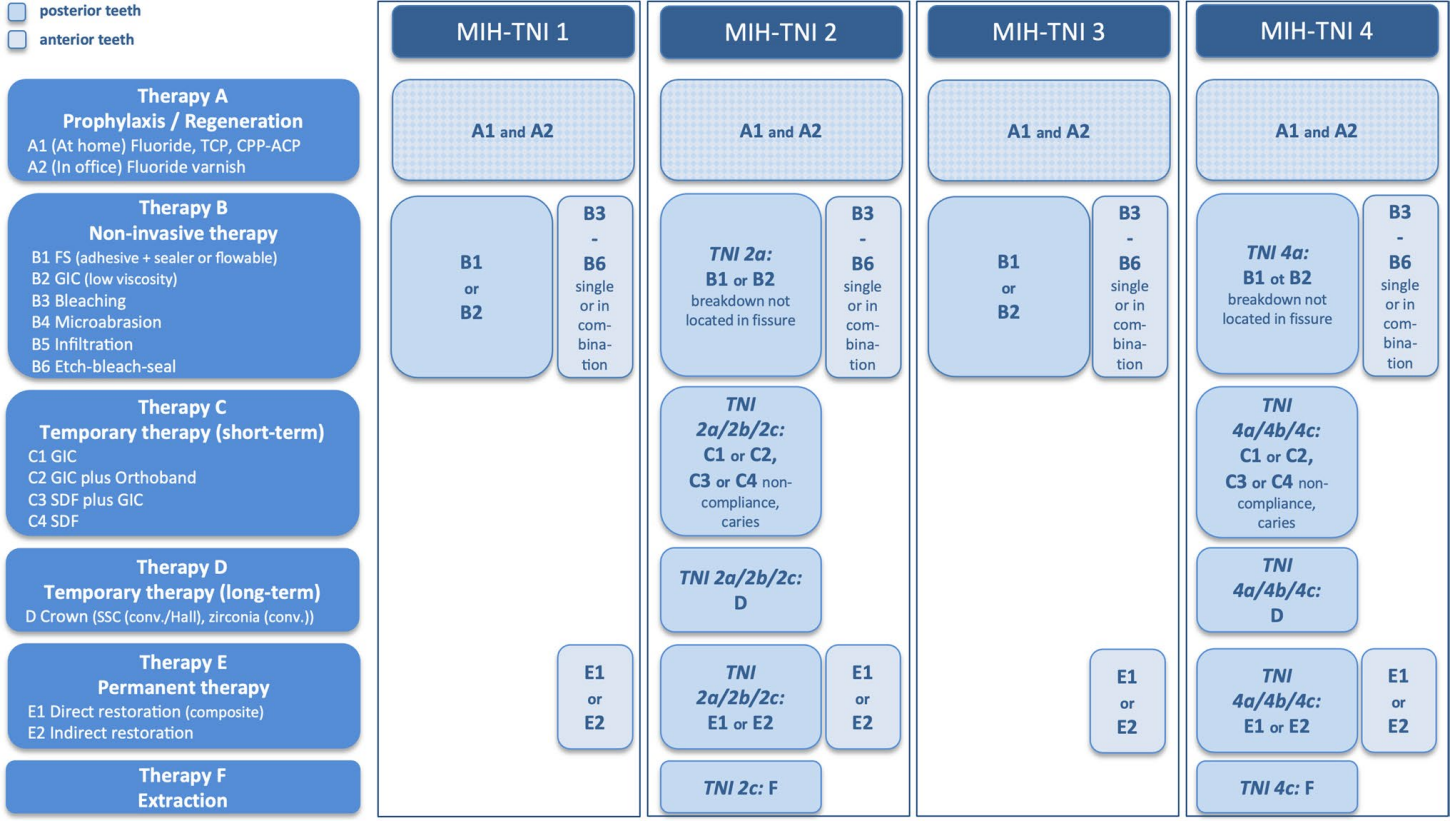
Treatment plan based on the MIH-TNI

MIH has been studied extensively in the last years (Lygidakis et al. 2022). Since 2016, knowledge about MIH has increased due to the availability of more clinical and laboratory studies. Other treatment approaches are now available, have been shown to be useful and therefore need to be included in an updated version of the Würzburg MIH concept. Therapy headings have also had to be reworded. For example, the new version of the flow chart now includes non-invasive strategies for molars, such as SDF, and treatment approaches for incisors. Furthermore, the two flow charts (low and high caries risk) have been merged into one chart (Fig. 1).

## Therapy A: prophylaxis/regeneration

Prophylaxis is important as MIH-affected children have a higher caries risk (Lygidakis et al. 2022). A recent study showed that the presence of MIH was associated with a 6.15 times higher prevalence of dental caries in first permanent molars (Oreano et al. 2023). Prophylaxis and regeneration include ´at home´ and ´in office´ approaches. Toothpastes containing fluoride (Ghanim et al. 2017) (possibly plus TCP) should be used twice a day. This may be accompanied by the additional use of CPP-ACP in a tray once a day (Baroni and Marchionni 2011). Topical fluoride varnish can be applied ‘in office’ 2–4 times per year depending on caries risk (Toumba et al. 2019). The need for prophylaxis is independent of the severity of the diagnosed TNI.

## Therapy B: non-invasive therapy

This section has been reworded. "Sealing" has been replaced by "non-invasive therapy" to also cover the treatment of incisors. For molars, non-invasive therapy approaches include sealants (Lygidakis et al. 2022). These can be either a fissure sealant or a flowable (both with pre-application of an adhesive (Lygidakis et al. 2009) and if the tooth is fully erupted) or a glass ionomer cement (if the tooth is not fully erupted). Incisors can be treated with bleaching (in adolescents) (B3) (Ghanim et al. 2017), microabrasion (B4) (Bhandari et al. 2019), infiltration (B5) (Marouane and Manton 2021; Altan and Yilmaz 2023), etch-bleach-seal technique (B6) [23] or a combination of these options.

## Therapy C: temporary therapy (short-term)

Therapy C has also been reworded. "Temporary restoration" has been replaced by "temporary therapy" to include other treatment modalities. Originally, this section only included short-term provisional treatment options using glass ionomer cement (Fragelli et al. 2015; Linner et al. 2020) without/with orthoband (Steffen and van Waes 2011). In the updated concept, the use of SDF (Seifo et al. 2019; Ballikaya et al. 2022) without/with GIC has been added.

## Therapy D: temporary therapy (long-term)

Therapy D has also been revised. The term "temporary restoration" has been replaced with "temporary therapy" as in Therapy C. In addition to stainless steel crowns (Kotsanos et al. 2005; Oh et al. 2020), the option of placing a zirconia crown (Talekar et al. 2023) has been added, as has the preparation technique for both crowns.

## Therapy E: permanent therapy

This part covers permanent restorations in the form of direct (composite) and indirect options (Sonmez and Saat 2017; Linner et al. 2020; Lygidakis et al. 2022). “Permanent restoration” has also been changed into “permanent therapy”.

## Therapy F: extraction

The therapy plan is completed with therapy F, extraction. In severe cases, when molars show massive post-eruptive breakdowns, the pulp is involved or dental abscesses are present, extraction is the treatment of choice (Lygidakis et al. 2022).

## Posterior teeth

### MIH-TNI 1

For MIH posterior teeth showing no breakdown and hypersensitivity (MIH-TNI 1), sealing therapy is considered the method of choice in addition to prophylaxis. If the tooth is fully erupted, this procedure should be carried out with a conventional fissure sealant or a flowable with the pre-application of an adhesive (Lygidakis et al. 2009). If the molar has not yet fully erupted, a temporary fissure sealant should be applied using a low viscosity glass ionomer cement.

### MIH-TNI 2

If MIH-TNI 2 is diagnosed in posterior teeth, the start of therapy depends on the location and size of the breakdown. If the substance loss is not located in the fissure and involves < 1/3 of the surface of the tooth, sealing therapy (B) may be the first step of treatment. However, if the loss of substance is located in the fissure or if the defect is > 1/3 or > 2/3, or if defects are found close to the pulp, the short-term temporary therapy (C) using a GIC with or without orthoband (C1, C2) is the therapy of choice, which can be converted to a definitive restoration (E, direct or indirect restoration) later (Sonmez and Saat 2017, Linner et al. 2020; Lygidakis et al. 2022). Thereby, indirect restorations should be considered if the child is older.

If the patient is non-compliant and caries is present, SDF can be used with or without the additional placement of a GIC (C3, C4) (Seifo et al. 2019; Ballikaya et al. 2022). Alternatively, a long-term temporary restoration in the form of a steel crown or a zirconia crown can be chosen (D) (Kotsanos et al. 2005; Oh et al. 2020; Talekar et al. 2023). The preparation technique can be conventional (both materials) or Hall (stainless steel crown, (Innes et al. 2007)). In addition, extraction should be considered as a long-term solution for TNI 2c at the appropriate time (Lygidakis et al. 2022). In this case, it is essential to consult with the orthodontist to determine the optimal time for extraction. If the time for extraction has not yet been reached, every effort should be made (therapy B, C and possibly even D) to preserve the MIH tooth until then.

### MIH-TNI 3

If there is no breakdown but hypersensitivity, sealing should be considered as initial therapy in posterior teeth to reduce pain. This can be done with a fissure sealant (B1) (Bekes et al. 2021, 2022). If the tooth has not fully erupted, sealing with a low viscosity glass ionomer cement (B2) can also be performed.

### MIH-TNI 4

If breakdown and hypersensitivity are present in posterior teeth, the patient follows the same steps as for MIH-TNI 2. Again, the size and the location of the defect is important. If the breakdown is minimal (TNI 2a) and not in the fissure, sealing can begin. However, if the defect is located in the fissure or if the defect is > 1/3 or > 2/3 in its extension or close to the pulp, then—as with MIH-TNI 2—short-term temporary therapy (C) with GIC (Fragelli et al. 2015; Linner et al. 2020) with or without orthoband is the approach of choice. As with TNI 2, if the patient is non-compliant and caries is present, SDF can be used with or without the additional placement of a GIC. (C3, C4) (Seifo et al. 2019). This temporary restoration can be converted to a permanent restoration (E) if the patient is compliant and a rubber dam can be achieved. This can be a direct or indirect restoration (Sonmez and Saat 2017; Linner et al. 2020; Lygidakis et al. 2022). Alternatively, the stainless steel or zircona crown is the temporary long-term restoration option (D) (Kotsanos et al. 2005; Oh et al. 2020; Talekar et al. 2023). In addition, extraction should also be considered as a long-term solution for TNI 2c at the appropriate time (Lygidakis et al. 2022).

## Anterior teeth

### MIH-TNI 1–4

There are many options, but not every hypomineralized anterior tooth needs to be treated from a dental point of view. Parents often want to act early in the interest of the child. However, it is not the parents but the child who should be asked about the existing pressure of suffering. Measurement of oral health-related quality (OHRQoL) might help to understand the child´s perspective (Shayestehpour et al. 2022) as it is known that anterior teeth affected by MIH can have an impact. In particular, problems related to social and emotional well-being have been described (Reissenberger et al. 2022). Treatment can improve the perception of oral health (Hasmun et al. 2020). However, young patients should be treated conservatively because of the large pulp chambers, high pulp horns and immature gingiva. In addition, a minimally invasive approach allows preservation of tooth structure for future restorative options (Lygidakis et al. 2022).

In addition to prophylaxis, anterior teeth can be treated with either bleaching (in adolescents) (B3) (Ghanim et al. 2017), microabrasion (B4) (Bhandari et al. 2019), infiltration (B5) (Marouane and Manton 2021; Altan and Yilmaz 2023), etch-bleach-seal technique (B6) (Prud'homme et al. 2017) or a combination of these options. However, it must be noted that independent of the technique chosen further investigations are required as well as improvement of material properties, and/or technical modifications in protocols before it can be fully recommended (Lygidakis et al. 2022).

Restorative techniques (E) may also be considered. With or without enamel removal, they can mask opacities of all shades and replace areas with post-eruptive breakdowns (Fayle 2003). However, indirect techniques should only be used in adolescents.

## Conclusions

The severity of hypomineralized MIH teeth and associated problems can vary widely. The Würzburg concepts provide an easy-to-use clinical index and a treatment plan based on it that can be used in daily practice. It also shows how to relieve the patient's pain in an emergency situation and how to implement an individualised long-term solution once the affected teeth have fully erupted. The updated Würzburg MIH Concept reaffirms Part 1, the use of the MIH-TNI. Part 2, the treatment plan, has been updated to include other available treatment approaches and has been expanded to include the treatment of anterior hypomineralized teeth. Further clinical studies should demonstrate the evidence of the concept.

## Data Availability

The paper does not include additional data.
